# Short term exposure to air pollution and mortality in the US: a double negative control analysis

**DOI:** 10.1186/s12940-022-00886-4

**Published:** 2022-09-06

**Authors:** Rongqi Abbie Liu, Yaguang Wei, Xinye Qiu, Anna Kosheleva, Joel D. Schwartz

**Affiliations:** Department of Environmental Health, Harvard T H Chan School of Public Health, 677 Huntington Ave, Boston, MA 02115 USA

**Keywords:** Air pollutant, Particulate matter, PM2.5, Ozone, Nitrogen dioxide, Mortality

## Abstract

**Rationale:**

Studies examining the association of short-term air pollution exposure and daily deaths have typically been limited to cities and used citywide average exposures, with few using causal models.

**Objectives:**

To estimate the associations between short-term exposures to fine particulate matter (PM_2.5_), ozone (O_3_), and nitrogen dioxide (NO_2_) and all-cause and cause-specific mortality in multiple US states using census tract or address exposure and including rural areas, using a double negative control analysis.

**Methods:**

We conducted a time-stratified case-crossover study examining the entire population of seven US states from 2000–2015, with over 3 million non-accidental deaths. Daily predictions of PM_2.5_, O_3_, and NO_2_ at 1x1 km grid cells were linked to mortality based on census track or residential address. For each pollutant, we used conditional logistic regression to quantify the association between exposure and the relative risk of mortality conditioning on meteorological variables, other pollutants, and using double negative controls.

**Results:**

A 10 μg/m^3^ increase in PM_2.5_ exposure at the moving average of lag 0–2 day was significantly associated with a 0.67% (95%CI: 0.34–1.01%) increase in all-cause mortality. 10 ppb increases in NO_2_ or O_3_ exposure at lag 0–2 day were marginally associated with and 0.19% (95%CI: −0.01-0.38%) and 0.20 (95% CI-0.01, 0.40), respectively. The adverse effects of PM_2.5_ persisted when pollution levels were restricted to below the current global air pollution standards. Negative control models indicated little likelihood of omitted confounders for PM_2.5_, and mixed results for the gases. PM_2.5_ was also significantly associated with respiratory mortality and cardiovascular mortality.

**Conclusions:**

Short-term exposure to PM_2.5_ and possibly O_3_ and NO_2_ are associated with increased risks for all-cause mortality. Our findings delivered evidence that risks of death persisted at levels below currently permissible.

**Supplementary Information:**

The online version contains supplementary material available at 10.1186/s12940-022-00886-4.

## Introduction

Globally, the burden of death attributable to fine particulate matter (PM_2.5_) is estimated to be more than 4 million annually, representing 7.6% of total global deaths [1, 2]. Short-term exposure to PM_2.5_ is associated with mortality from all-causes [3–5], stroke [6], asthma [7, 8], and chronic obstructive pulmonary disease [9–11]. Exposure to O_3_ and NO_2_ has also been linked to chronic respiratory diseases, impaired lung function, and all-cause mortality [12–16].

However, previous studies of the acute effect of PM_2.5_ have been restricted to well-monitored metropolitan areas where the population is large enough to power the studies [17, 18]. Many time-series which examined the acute effect of O_3_ and NO_2_ on daily deaths had the same limitations [14, 15, 19]. Hence the effects in rural areas and unmonitored areas have been under-examined. In addition, these time-series studies assigned the same exposure to everyone in the same city, entailing limited spatial resolution and considerable exposure error. Fewer studies have examined all three of these pollutants together, with only one using causal modeling methods [20], and studies below the previous World Health Organization Air Quality Guidelines (WHO AQG) [21] are less common.

In this study, we studied the entire population of all ages in seven US states, use census tract or finer exposure data, and examine the lag structure between short-term air pollution exposure and all-cause and cause-specific mortality using a time-stratified case-crossover design. The study population covered states in the Midwest and Eastern U.S. between 2000 and 2015, with over 3 million deaths. We have also implemented several causal methods, specifically negative exposure controls and negative outcome controls to provide more evidence for the causality of any associations.

## Methods

### Study population

This study used non-accidental mortality data across seven states of the US: Georgia, Indiana, Kansas, Massachusetts, Michigan, New Jersey, and Ohio. Death certificate data were obtained from each state’s department of health and included date of death, age, sex, race, education, marital status, the cause of death, and either the census tract number or the latitude and longitude of the residential address at the time of death. The study outcomes were all-cause and cause-specific mortality due to cardiovascular disease (ICD-10: I00 to I99) and respiratory disease (ICD-10: J00 to J99).

### Air pollution exposures and meteorological covariates

Daily concentrations of PM_2.5_, O_3_, and NO_2_ at 1 km x 1 km grid cells in the contiguous US were predicted using a well-validated hybrid prediction model that incorporates satellite remote sensing, chemical transport models, meteorological variables, and land-use terms, with out-of-sample predicted R^2^ of 0.86, 0.90, and 0.79 respectively [22, 23]. With this model, predictions were generated across the entire contiguous US. Temperature and absolute humidity were retrieved from Phase 2 of the North American Land Data Assimilation System, and daily mean values were determined for each 12 kmx12 km grid across the continental United States [24]. For each individual decedent, the daily mean PM_2.5_, daily 8-hour maximum ozone (O_3_), daily 1-hour maximum nitrogen dioxide (NO_2_), daily mean temperature, and daily mean absolute humidity were assigned.

### Study design

We utilized a case-crossover design with “case day” defined as the date of death, and “control day” defined as the same day of the week within the same month and year where death did not occur. “Control day” was chosen bidirectional time stratified (i.e. both before and after the case day, but in the same month) to control for confounding by time trend. For each individual, we compared daily air pollution exposure on the case day to control days. By virtue of the study design, individuals serve as their own controls and any subject-level covariates that remain constant on case and control days (i.e., age, gender, race, socioeconomic status, comorbidities, smoking history, cholesterol levels, diet, obesity, etc.), as well as any seasonal and sub-seasonal patterns, are controlled for by design. We further used both a negative exposure control and a negative outcome control to deal with the potential for unmeasured confounders [25].

### Statistical analysis

We used conditional logistic regression models to assess the associations between acute air pollutant exposures and mortality. Based on prior studies, for each exposure, we assigned a moving average of the same data and two previous days to each decedent on the case and control days. Temperature was included as same day temperature, a moving average of lag1–3, and an additional quadratic term. Humidity was included as same day humidity and a moving average of lag1–3. Exposures after the death (lead) were included as negative exposure controls. They clearly cannot have caused the death, but if there is an omitted time-varying confounder that is correlated with the air pollution on the day of death, it is likely also correlated with the pollutant on the following day. Hence control for lead 1-day can at least partially control for that omitted confounder and identify the likely direction of bias. If the coefficient relating the omitted confounder to pollution is the same on the day of death and the subsequent day, then the unconfounded estimate of the true effect would be the difference between the coefficient of exposure on death and the coefficient of lead 1 exposure on death. Given the two exposures differ by a single day, this is a reasonable, but not certain assumption. Further, since that estimate controls for unmeasured confounding, it would be a causal estimate.

The lag periods selected for inclusion are based on epidemiologic literature reporting evidence of immediate effects of air pollution on mortality (i.e., within a few days after pollution exposure) [26, 27]. We evaluated the effect of each pollutant in single-pollutant models, double-pollutant models, and three-pollutant models. We estimated the percent increase in mortality and its 95% confidence intervals (CIs) associated with each 10 μg/m^3^ increase in the exposure of PM_2.5_ or 10 ppb increase in exposures to O_3_ and NO_2_.

**Fig. 1:**
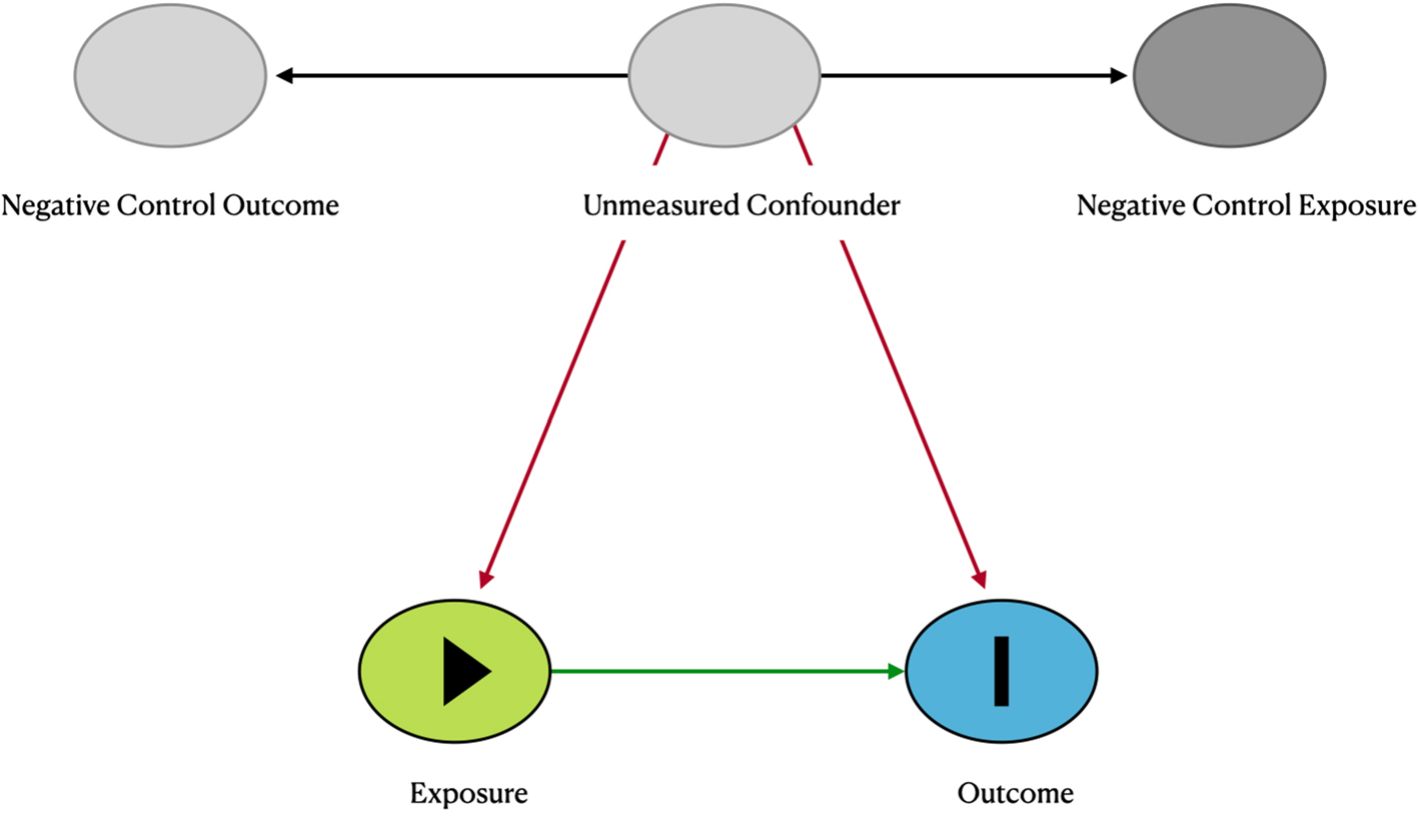
DAG for the double negative control Scenario

In addition to the negative exposure control, we separately analyzed deaths due to non-alcoholic fatty liver disease (NAFLD), which served as a negative outcome control to examine potential omitted confounding [28]. Again, if we unexpectedly find an association of exposure with the negative outcome control, that would indicate the presence of an omitted confounder that was associated with both. Finally, using a two-stage approach similar to two-stage least squares, one can relax the assumption of equal association between the omitted confounder(s) and both exposure and negative control exposure and obtain a bias corrected causal estimate, under the usual assumptions for causal models (SUVTA). Essentially, the expected value of the negative control outcome can be used as a surrogate for the omitted confounder(s) and by controlling for it in the model with the negative exposure control we can obtain bias corrected estimates using this double negative control. The details are shown in Additional file 6. A DAG for this scenario is included in Fig. 1. As seen in this figure, there is a backdoor path between the negative control exposure and both outcome and exposure through unmeasured confounder U, and similarly for the negative control outcome. It is by making use of these two associations that, under appropriate assumptions, one can indirectly control for U.

We repeated the analyses restricting to deaths with exposure levels below the 2020 World Health Organization Air Quality Guidelines (WHO AQG) for each pollutant to examine whether the associations persisted at levels currently permissible (25 μg/m^3^ for PM_2.5_, 100 μg/m^3^ for O_3_, 200 μg/m^3^ for NO_2_).

### Effect modification analysis

To identify potentially susceptible populations, we examined modifications among subgroups of sex (male and female), race (White, Black, and Other), age (≤45, 45–65, 65–75, and ≥ 75 years), education (less than, equal to, or greater than high school) and urbanicity (urban, rural). Population density for each census tract was calculated using the total population and land area, and urbanicity was defined based on whether census tract population density exceeds the 25th percentile of the overall density of the entire study population.

### Sensitivity analysis

Sensitivity analyses were conducted to examine the robustness of our results. First, we evaluated different lag periods for temperature and humidity and chose the estimate of moving average in the final model based on the most robust estimates of individual lag patterns. We also included an interaction term between temperature and state.

All analyses were done in the statistical environment R4.0.3 [29], with the “survival” package (version 3.2–7) to fit the conditional logistic regression [30]. This study was approved by the institutional review board at Harvard T.H. Chan School of Public Health.

## Results

### Variable distribution and descriptive statistics

A total of 3,063,192 deaths were identified between 2000 and 2015 with a complete record of the date of death as well as corresponding geographical coordinates. Table 1 presents the summary statistics for the total population examined and for each state. Among all subjects who died during the study period, 46.9% were male and 12.8% were of the non-white race. The mean age at death was 75.6 years, ranging from 1.9 to 117.0 years, with 77% of the cases occurring in people 65 years or older. Of all deaths, 1,053,304 (34.4%) deaths were from cardiovascular diseases, and 323,309 (10.6%) deaths were from respiratory diseases.

**Table 1:** Descriptive characteristics and event day exposures from 2000 to 2015 in the US and in each state included in the study

	Total	OH	MA	NJ	GA	KS	IN	MI
	***N*** = 3,063,192	***N*** = 691,180	***N*** = 986,257	***N*** = 355,231	***N*** = 311,146	***N*** = 63,462	***N*** = 99,035	***N*** = 556,881
**Sex**								
Male	46.9%	47.3%	45.9%	46.2%	48.0%	46.9%	47.3%	48.0%
Female	53.1%	52.7%	54.1%	53.8%	52.0%	53.1%	52.7%	52.0%
**Age (years)**	75.6 (18.1)	75.1 (16.5)	77.4 (20.4)	75.9 (16.2)	72.1 (18.1)	76.4 (17.2)	74.5 (16.8)	74.9 (16.6)
**Race**								
White	87.2%	88.4%	92.8%	84.3%	71.7%	92.0%	91.8%	84.7%
Black	10.8%	10.9%	4.0%	12.6%	27.3%	4.8%	7.6%	13.6%
Other	2.0%	0.7%	3.1%	3.2%	1.0%	3.2%	0.6%	1.6%
**Education**								
< HS^a^	21.2%	24.4%	17.8%	24.5%	11.7%	24.4%	15.8%	26.9%
HS	44.6%	49.0%	51.2%	49.9%	14.3%	42.1%	23.9%	45.0%
> HS	25.0%	23.9%	29.9%	24.9%	11.5%	32.3%	11.0%	26.6%
**Urbanicity**								
Urban	75.1%	70.6%	83.5%	91.6%	67.2%	32.8%	41.3%	68.8%
Rural	24.9%	29.4%	16.5%	8.4%	32.8%	67.2%	58.7%	31.2%
**Case Day Exposure**								
PM_2.5_ (*μ*g/m^3^)	10.4 (6.15)	11.8 (5.90)	8.66 (5.67)	11.7 (7.36)	11.8 (5.45)	9.71 (4.74)	13.0 (6.60)	9.63 (5.90)
O_3_ (ppb)	37.7 (11.0)	38.0 (10.5)	37.0 (11.0)	37.2 (12.0)	41.3 (12.6)	38.0 (10.6)	38.2 (13.2)	36.6 (8.52)
NO_2_ (ppb)	21.2 (12.1)	18.4 (10.1)	22.4 (11.8)	31.9 (13.4)	15.3 (10.7)	16.3 (10.0)	17.5 (9.95)	20.3 (10.8)
Temperature (Kelvin)	284 (10.3)	284 (10.4)	283 (9.61)	284 (9.63)	290 (9.21)	285 (11.2)	284 (11.2)	282 (10.7)
Humidity (g/cm^3^)	0.0073 (0.0045)	0.0076 (0.0045)	0.0068 (0.0042)	0.0076 (0.0046)	0.0095 (0.0047)	0.0077 (0.0048)	0.0075 (0.0046)	0.00667 (0.0042)

^a^Definition of abbreviations: HS=High school

For sex, race, and education, data were presented as a percentage to the total. For age and case-day exposure, data were presented as mean (standard deviation)

Table 1 also presents the distribution of air pollutants and meteorological covariates on case days. The mean daily ambient air pollutant concentrations over the study period were 10.3 μg/m^3^ for PM_2.5_, 37.7 ppb for O_3_, and 21.2 ppb for NO_2_. Concentrations varied year-to-year and between states, likely due to meteorology and wind patterns, and spatial variability in local sources of pollution.

### Results for single, double and multi-pollutant air pollutant models

**Fig. 2:**
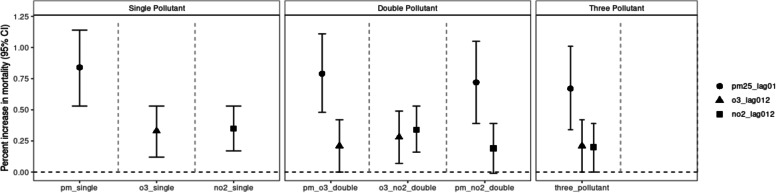
Effect of air pollution on all-cause mortality in single, double and three pollutant models

Figure 2 presents the result for percent increase in all-cause mortality in single-, double-, and three-pollutant models. Supplementary Table 4 shows the detailed results. Individually, all three pollutants were significantly associated with an increase in all-cause mortality. Upon controlling for either O_3_ or NO_2_ in double pollutant models, and for both in the three-pollutant model, the effect of PM_2.5_ attenuated slightly, but remained significant. The effect of O_3_ and NO_2_ attenuated to marginally significant after adjusting for PM_2.5_ in the two pollutant models, and both became only marginally significant in the three-pollutant model.

Tables 2 and 3 presents the results of the analyses for all-cause mortality using the moving average of air pollutants and adjusting for all other pollutants, temperature, absolute humidity, and the leads of each pollutant. In the three-pollutant model, the percent increases for all-cause mortality associated with each 10 μg/m^3^ increase of PM_2.5_ exposure at lag 0–2 day, and 10 ppb increase in NO_2_ exposure at lag 0–2 day were 0.73% (95%CI: 0.38–1.08%), and 0.19% (95%CI: −0.01-0.38%), respectively. Each 10 ppb increase in O_3_ exposure at lag 0–2 day was associated with a 0.20% (95%CI: −0.01-0.41%) increase in all-cause mortality, although the association was only marginally significant (p < 0.06) for the gaseous pollutants. For PM_2.5_, we found larger effect sizes for respiratory deaths, at 1.16% (95%CI: 0.00–2.35%) per 10 μg/m^3^ increase. PM_2.5_ was also significantly associated with deaths from cardiovascular causes (Tables 2 and 3, Fig. 2). No significant associations were seen for NO_2_ or O_3_ with the specific causes of death, although there was a marginal association of NO_2_ with respiratory deaths. We saw no significant association of any exposure with the negative outcome control.

**Table 2:** Estimated percent increase in all-cause and cause-specific mortality with increases in PM_2.5_, O_3_, and NO_2_ in baseline model, two stage causal model, and low exposure model

		PM2.5 (***μ***g /m^**3**^)			O3 (ppb)			NO2 (ppb)		
*Model*	*Cases (n)*	*%*	*95% CI*	*p*	*%*	*95% CI*	*p*	*%*	*95% CI*	*p*
*Three pollutant Model*	*13,474,216*	0.73	(0.38, 1.08)	<0.01	0.20	(−0.01, 0.41)	0.06	0.19	(−0.01, 0.38)	0.06
*With two Stage Causal Model*	*13,474,216*	0.68	(0.33, 1.03)	<0.01	0.30	(0.12, 0.48)	<0.01	0.12	(−0.02, 0.26	0.09
*Low Exposure*^a^	*11,919,986*	0.73	(0.38, 1.08)	<0.01	0.23	(−0.02, 0.48)	0.08	0.19	(−0.01, 0.39)	0.07
*Cardiovascular*^b^	*1,053,304*	0.79	(0.18, 1.40)	0.01	0.22	(−0.15, 0.59)	0.26	−0.13	(−0.48, 0.22)	0.47
*Respiratory*^b^	*323,309*	1.16	(0.00, 2.35)	0.04	0.41	(−0.33, 1.15)	0.28	0.73	0.00, 1.46)	0.05

*Values are percent increase (95% CI) for 10 μg/m*^*3*^*increase in PM*_*2.5*_*, 10 ppb in O*_*3*_*, and 10 ppb in NO*_*2*_*. All models were adjusted for temperature and absolute humidity. Lag periods for all models were lag0–1 for PM*_*2.5*_*, lag0–2 for O*_*3*_*, and lag0–2 for NO*_*2*_

^a^*The low exposure model analysis had the same model specifications as the baseline model analysis and was restricted to days with PM*_*2.5*_*below 25 μg/m*^*3*^*, O*_*3*_*below 50 ppb, and NO*_*2*_*below 106.4 ppb*

^b^*Mortality due to cardiovascular disease (International Classification of Disease, 10th edition [ICD-10] codes I00 to I99) and respiratory disease (ICD-10 codes J00 to J99)*

**Table 3:** Association of negative control exposure with outcome

	PM2.5 lead (***μ***g /m^**3**^)			O3 lead (ppb)			NO2 lead (ppb)		
*Model*	*%*	*95% CI*	*p*	*%*	*95% CI*	*p*	*%*	*95% CI*	*p*
*Three pollutant Model*	−0.36	(−0.67, −0.11)	<0.01	0.19	(0.01, 0.37)	<0.05	−0.047	(−0.23,0.12)	0.55
Low Exposure^*^	−0.32	(−0.63, 0.003)	0.05	0.27	(0.07, 0.47)	<0.01	−0.05	(−0.23, 0.14)	0.64
*Cardiovascular*^*†*^	−0.49	(−0.97, −0.01)	<0.05	0.26	(−0.05, 0.56)	0.10	0.14	(−0.16, 0.44)	0.37
*Respiratory*^*†*^	−0.38	(−1.28, 0.52)	0.41	−0.57	(−0.13, −0.003)	<0.05	−0.42	(−0.99, 0.16)	0.16

*Values are percent increase (95% CI) for 10 μg/m*^*3*^*increase in PM*_*2.5*_*, 10 ppb in O*_*3*_*, and 10 ppb in NO*_*2*_*. All models were adjusted for temperature and absolute humidity. Lag periods for all models were lag0–1 for PM*_*2.5*_*, lag0–2 for O*_*3*_*, and lag0–2 for NO*_*2*_

*The threshold model analysis had the same model specifications as the baseline model analysis and was restricted to days with PM_*2.5*_*below 35 μg/m*^*3*^*, O*_*3*_*below 70 ppb, and NO*_*2*_*below 100 ppb*

*†Mortality due to cardiovascular disease (International Classification of Disease, 10th edition [ICD-10] codes I00 to I99) and respiratory disease (ICD-10 codes J00 to J99)*

### Restriction to effects below standard

**Fig. 3:**
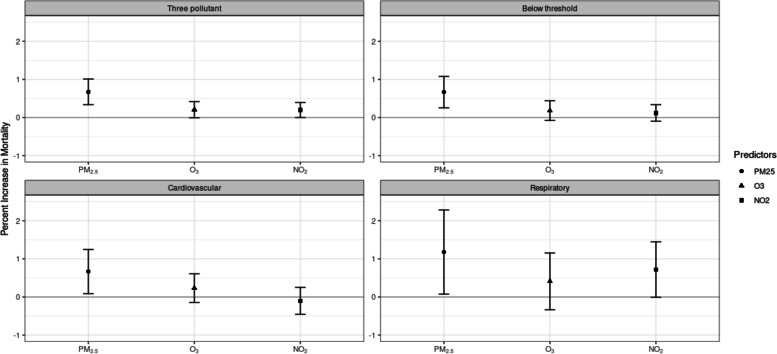
Estimated percent increase in all-cause and cause-specific mortality with increases in PM_2.5_, O_3_, and NO_2_ in baseline model and low exposure model

Of all case and control days, 98.0% days had PM_2.5_ levels below the WHO AQG standard of 25 μg/m^3^, 89.5% days had ozone levels below the standard of 50 ppb (100 μg/m^3^), and 100% days had NO_2_ levels below the standard of 106.4 ppb (200 μg/m^3^). When restricted to days with PM_2.5_ exposure below standards, the results remained unchanged and significant for PM_2.5_ (Table 2 and 3, Fig. 3). The results for O_3_ and NO_2_ also were little changed and remained marginally significant.

### Effect modification

**Fig. 4:**
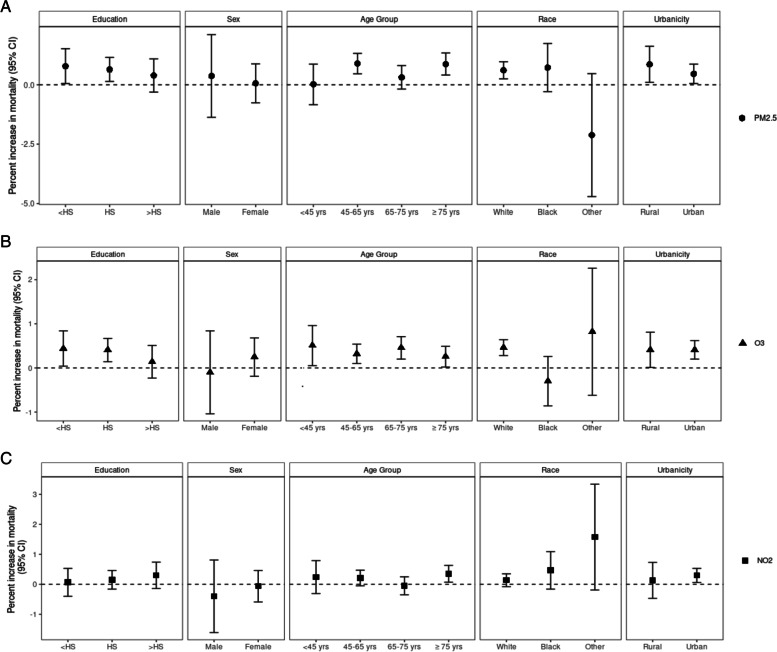
Percent increase in all-cause mortality associated with each air pollutants in subgroups of effect modifiers

Figure 4 and presents the effect of each air pollutant among subgroups of education, sex, age group, race, and urbanicity. Although we did not observe significant effect modification (Supplementary Table 1), there was a trend of decreasing effect size for increasing education for PM_2.5_ and O_3_, of larger effects of PM_2.5_ in less densely populated locations, and of lower effects of O_3_ but larger effects of NO_2_ in Blacks.

### Sensitivity analysis

Temperature and absolute humidity on lag days 1 to 3 had robust associations with all-cause mortality (Supplementary Table 2) and the moving averages of these days were selected for the final model, along with terms for same-day temperature and humidity. We also added a non-linear quadratic term for same-day temperature in the final model. In addition, we performed a sensitivity analysis with a separate temperature effect for each state (Supplementary Table 3). The PM_2.5_ effect estimate was little changed in this sensitivity analysis, but both the O_3_ and NO_2_ associations because statistically significant.

### Causal modeling

The greatest threat to the validity of environmental epidemiology studies is omitted confounding. Negative outcome controls are a form of causal modeling that captures confounding by unmeasured covariates that are expected to be predictors of the both the outcome and the negative control outcome, and no association was found between any of the pollutants and the negative control outcome. Similarly, the negative exposure control captures confounding by time varying confounders that were not measured, and this was incorporated in the main analysis. The negative control exposure was negatively and significantly associated with mortality for PM_2.5_, negative but insignificant for NO2, and positive and significant for O_3_ (Supplementary Table 2). When we used these estimates to correct for omitted confounders assuming they have the same correlation with exposure and exposure in the subsequent day (Tables 2 and 3), the effect sizes increased for PM_2.5_ and NO_2_ to 1.09% (95% CI 0.74, 1.45) and 0.23% (95% CI 0.03, 0.44) respectively, while the effect size for ozone was diminished at 0.00% (95% CI (−0.26, 0.27). The two-stage model controlling for the expected value of the negative outcome produced similar results to the original model for PM_2.5_, larger results for ozone, and smaller effect estimates for NO_2_.

## Discussion

In this study, we conducted a time-stratified case-crossover analysis for major air pollutants using spatially resolved exposure data, estimating the associations of short-term PM_2.5_, O_3_, and NO_2_ exposures with mortality for the entire population of seven US states at the individual level, which covered over 3 million deaths that occurred between 2000 to 2015. These estimates were not restricted to major cities but include smaller cities and rural areas. Exposure was assigned either as the concentration in the 1 km grid cell that contained the home address of the decedent, or the census tract of the decedent, which is a much finer spatial resolution that most preceding studies. Moreover, because we used spatio-temporal exposure models differences in the temporal pattern of exposure by geography were incorporated, which has not been the case for city-wide time series studies.

We found an independent and significant effect for PM_2.5_ and a marginal one for NO_2_, where a 10 μg/m^3^ and 10 ppb increase was significantly associated with a 0.73 and 0.19% increase in the risk of all-cause mortality, respectively. The association with O_3_ (0.20) was also marginally significant in the three-pollutant model. The association for PM_2.5_ remained significant when restricting the analysis to days with pollutant levels lower than the WHO AQG [31] (25 μg/m^3^), indicating that current standards are not sufficient to protect the general population. Importantly, we incorporated a double negative control strategy to protect against confounding by omitted variables. We controlled for negative exposure control (exposure after death) in the main analysis, which would capture any omitted covariate that was correlated with both air pollution before and after the death and mortality. In addition, we saw no association of any pollutant with the negative outcome control (mortality due to NAFLD), which would capture any time varying covariate that is associated with deaths from any cause (including NAFLD). Finally, we used a two-stage approach that treats the expected NAFLD cases as a surrogate for the omitted confounders, in our model for all-cause mortality. The effect size for PM_2.5_ was little changed, increased and became significant for O_3_, and decreased for NO_2_. The case-crossover design itself controls by matching for slowly varying individual and neighborhood covariates. Together, these suggest that the PM2.5 association is robust to control for other pollutants and omitted confounders, and the two-stage and negative control analyses strongly suggest a causal association. The NO_2_ and O_3_ results are more mixed with mostly marginal associations in multipollutant models and more indication of omitted confounding, albeit of unsure direction of bias. However, in the models with state specific temperature effects, both gaseous pollutants were significant.

Although other publications have investigated the effect of air pollutants utilizing a case-crossover design [32–34], none was on the scale in terms of area and age coverage comparable to the present study. In addition, our high exposure resolution has not yet been provided by existing literature. Case-crossover analyses have mostly assigned the same exposure to all inhabitants in a city or metropolitan area. In contrast our exposure was assigned at the individual address or census tract. Hence, our models greatly reduce exposure error. Of course, while the exposure models were very good, exposure error still remains, and can still induce bias in effect sizes. A recent simulation study of multipollutant measurement error reported that the bias was almost always toward the null [35]. A case-crossover study of Medicare participants by Di et al. used spatially resolved air pollution at the ZIP code level [22] which had a coarser resolution as compared to our census tract level exposure (about one-third of the population of a ZIP code) or 1 km exposure. Our effect estimates for PM_2.5_ and O_3_ were lower than that of Di’s (0.73 and 0.20% respectively), but we also adjusted an additional air pollutant NO_2_ as well as incorporated negative exposure controls, and negative outcome controls, and two-stage methods. The observed associations between PM_2.5_ and mortality were robust to adjustment by co-pollutants and weather variables. In addition, while Di restricted the study to the US Medicare population of people 65 years and older, our study included people of all ages, providing increased generalizability.

The effect of PM_2.5_ was in agreement with those obtained by a study across 112 US cities from 1999–2005, which reported a 0.98% (95% CI: 0.75–1.22%) increase in mortality with each 10 μg/m^3^ increase in PM_2.5_ [36]. Although our estimation for O_3_ was only marginally significant, the estimate was on par with that observed in a study of 48 US cities, which found a 0.3% (95% CI: 0.2–0.4%) increase in total mortality with each 10-ppb increase in O_3_ [15]. However, similar to other previous US studies [18, 37–41], those daily air pollutant exposure data were obtained from local ambient monitoring stations. As a result, all individuals residing in the metropolitan area were assigned the same exposure, leading to substantial measurement error. In comparison, the present study did not use central monitors, thereby providing a finer resolution and more accurate exposure data for all individuals, including individuals living in smaller cities, rural communities or unmonitored areas that would be misclassified or not included in earlier time-series studies. We observed a larger, although insignificantly different, effect for PM_2.5_ and NO_2_ in rural areas as compared to urban areas, suggesting the need for improved rural monitoring to contrast the adverse effect in urban versus rural regions, and the need to examine sources of rural vulnerability.

Findings from this study were also consistent with the effect sizes of PM_2.5_ observed in other countries [42–44]. However, our estimates for PM_2.5_ were higher than the 0.22% increase in 272 Chinese cities [45] and the 0.55% increase in 10 Mediterranean metropolitan areas [46]. Those regions have higher PM_2.5_ concentrations, and the lower effect sizes may be due to a nonlinear dose-response, with lower slopes at high concentrations, which has been reported previously [47]. On the other hand, our estimates for NO_2_ were lower than the 0.9% increase in previously reported studies [19], although that study did not control for O_3_ and PM_2.5_. These discrepancies may also be partly explained by differences in population structure, the number of cities, age category, and air pollutant measurement method involved. The marginal insignificance of the O_3_ association when controlling for NO_2_ should be treated cautiously, since NO_2_ has a complex association with O3, serving as a driver of photochemistry but also a marker for NO quenching in more heavily trafficked areas. This can create a complex confounding pattern that can lead to effect transfer across the two pollutants.

The WHO AQG daily standards were until recently 25 μg/m^3^ for PM_2.5_, 50 ppb for O_3_, and 106.4 ppb for NO_2_. In comparison, the United States has a less restrictive standard for PM_2.5_ and NO_2_ (35 μg/m^3^ for PM_2.5_, 70 ppb for O_3_, and 100 ppb for NO_2_). When restricting the analysis to a PM_2.5_ concentration below the WHO standards, its effect size remained the same. The EPA recently proposed to maintain the current national particulate matter standards due to insufficient evidence for effect at lower concentrations [48]. Our findings showed that even at levels below the standards, PM_2.5_ pollution is significantly associated with an increase in daily mortality rates, including after incorporation of multiple causal modeling methods.

In addition to all-cause mortality, we also found a significant association with cardiovascular and respiratory mortality for PM_2.5_. Exposure to air pollution has been consistently associated with death due to chronic obstructive pulmonary disease (COPD), death due to pneumonia, as well as emergency room visits for asthma [14, 15, 19, 49], and our estimates for respiratory mortality are in line with previously reported estimates. Many studies have reported associations between exposure to PM_2.5_ and cardiovascular deaths [19, 50] and provided evidence that these disease processes can be mediated through a combination of inflammatory, autonomic, and vascular changes [51, 52].

Profound racial and socioeconomic disparities in PM_2.5_ exposure have been well documented in prior studies, where the burden of death associated with PM_2.5_ exposure was disproportionately borne by the elderly [38, 53] and people of races other than white [54, 55]. Our effect modification analysis suggested a slightly elevated, although insignificantly different, association between PM_2.5_ and all-cause mortality among females, people of lower educational attainment, those residing in rural areas, and people of Black race. This is in addition to the effects of higher exposure in minorities. Greater attention is needed to address the issue faced by minorities who might also be least equipped to deal with the adverse health consequences of air pollution.

Attention has recently focused on causal methods of analysis for observational data. Causal modeling seeks to mimic a randomized controlled trial by making exposure independent of all confounders but can fail if there are omitted confounders. Case-crossover analyses, by matching each person to themselves, on a nearby day without the event make exposure independent of all fixed or slowly changing individual covariates by design, and hence render exposure independent of many unmeasured confounders. In addition, we used negative exposure and outcome controls to capture omitted time-varying confounders, and a two-stage regression model to control for unmeasured, time-varying confounders. These methods strengthened the evidence for a causal association between air pollution and daily mortality.

This study has several limitations. First, there is a lack of data differentiating exposure at residence and exposure elsewhere. However, in this study, 77% of the deaths occurred in people over the age of 65 and we, therefore, expected little workplace or commuting exposure, and a higher relevance for residential exposure [56]. As a result, the extent of misclassification was reduced. Moreover, the National Human Activity Pattern Survey in the U.S. reported that U.S. adults spent 69% of their time at home and 8% of the time immediately outside their home [57]. Second, we did not have individual data on behavioral factors, medication, and specific health histories or treatments. By design, these cannot be confounders, but this limited our ability to investigate potential modifications by these characteristics. Third, we did not investigate potential confounding by other co-pollutants such as sulfur dioxide (SO_2_) and carbon monoxide (CO). However, the levels of SO_2_ and CO are low in the US [58, 59]. In addition, Dominici et al. [60] adjusted for all O_3_, NO_2_, SO_2_, and CO but found no change in the magnitude of the effect between particular matter and mortality, suggesting there is little evidence that the effect of particulate matter is confounded by the additional pollutants. Finally, while our exposure models were good, they were not perfect in estimating exposure at 1 km resolution. Further, the exposure error in the models varied spatially, which may account for the lack of finding of interactions with spatially varying effect modifiers.

Despite its limitations, the study adds to our understanding of the effect of short-term air pollution exposure. The most important strength of this study is the high resolution of exposure data covering the multiple states, even in areas without air monitoring stations. This provided accurate estimates of daily levels of air pollution and meteorological conditions, allowing us to examine the entire population of these states instead of only larger cities, and reduced exposure misclassification compared to prior studies with a central-monitor approach. Second, our analysis on the whole population of seven US states avoids potential selection bias and ensures the generalizability of the results. Finally, we used several causal modeling techniques, including negative exposure and negative outcome controls to increase the likelihood of a causal association.

## Conclusions

In this analysis of the entire population in seven US states with over three million deaths, we found that short-term exposures to PM_2.5_, O_3_, and NO_2_ were individually associated with an increased risk of all-cause mortality. The effect of air pollution persisted even at low ambient concentrations, suggesting that the current daily standards may need to be revised to reduce the global burden of mortality due to air pollution. The use of multiple causal techniques increases the likelihood of causal relationships between the short-term air pollution exposures and mortality.

## Supplementary Information

**Additional file 1: Figure E1.** Percent increase in cause-specific mortality for PM_2.5_, O_3_, and NO_2_ using the single-lag model.

**Additional file 2: Supplementary Table 1.** Incremental effect modification.

**Additional file 3: Supplementary Table 2.** Single lag models for temperature and absolute humidity.

**Additional file 4: Supplementary Table 3.** Effects of Negative Exposure Controls.

**Additional file 5: Supplementary Table 4.** Effects (95% CI) in Single and Double pollutant models.

**Additional file 6.** Use of negative outcome and negative exposure controls to obtain estimated effects corrected for bias by omitted confounders.

## Data Availability

The air pollution datasets and R code generated during and/or analysed during the current study are available from the corresponding author on reasonable request. The health datasets are available from the Departments of Health of the respective States.
