# Supplementary figures and images for: Short term exposure to air pollution and mortality in the US: a double negative control analysis
Source: Environ Health. 2022 Sep 6;21:81. doi: 10.1186/s12940-022-00886-4 (PMC9446691; doi:10.1186/s12940-022-00886-4)

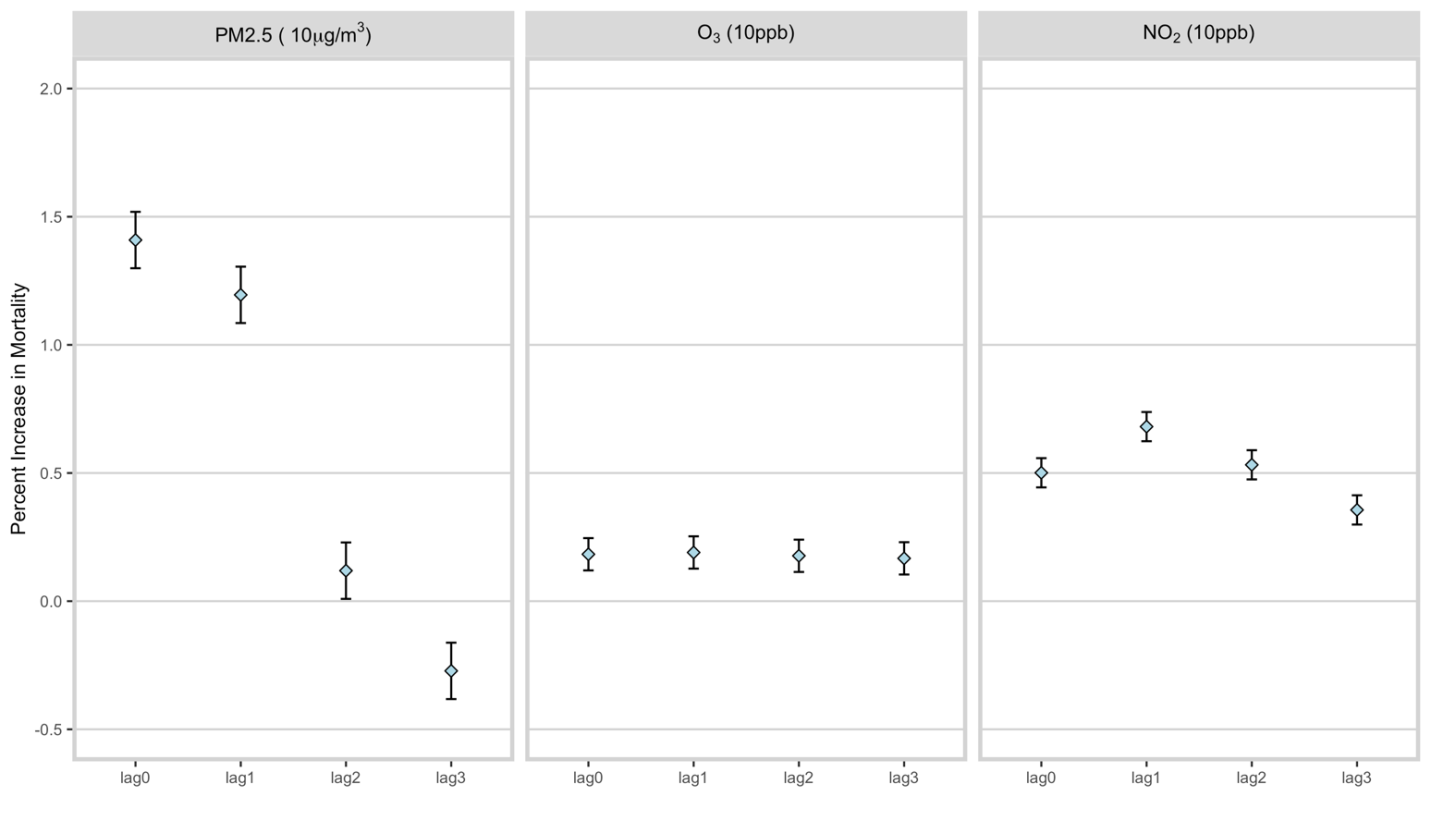

Supplement: Supplementary file 1 — Additional file 1: Figure E1. Percent increase in cause-specific mortality for PM2.5, O3, and NO2 using the single-lag model. [file 12940_2022_886_MOESM1_ESM.docx]
