# Supplementary material for: Short term exposure to air pollution and mortality in the US: a double negative control analysis
Source: Environ Health. 2022 Sep 6;21:81. doi: 10.1186/s12940-022-00886-4 (PMC9446691; doi:10.1186/s12940-022-00886-4)
Supplement: Supplementary file 2 — Additional file 2: Supplementary Table 1. Incremental effect modification. [file 12940_2022_886_MOESM2_ESM.docx]

|  | *Predictors^†^* | **PM_2.5_ (µg/m3)** | | | **O_3_ (ppb)** | | | **NO_2_ (ppb)** | | |
| --- | --- | --- | --- | --- | --- | --- | --- | --- | --- | --- |
|  |  | *%* | *95% CI* | *p* | *%* | *95% CI* | *p* | *%* | *95% CI* | *p* |
| Education | HS | -0.07 | (-0.40, 0.25) | 0.653 | 0.00 | (-0.10, 0.11) | 0.970 | -0.01 | (-0.17, 0.16) | 0.938 |
|  | >HS | -0.10 | (-0.46, 0.27) | 0.612 | 0.00 | (-0.12, 0.11) | 0.943 | -0.01 | (-0.20, 0.17) | 0.897 |
| Age group | 45-65 | 0.02 | (-0.62, 0.66) | 0.952 | 0.02 | (-0.19, 0.23) | 0.855 | 0.04 | (-0.29, 0.37) | 0.826 |
|  | 65-75 | -0.01 | (-0.65, 0.64) | 0.983 | 0.01 | (-0.19, 0.22) | 0.896 | 0.07 | (-0.26, 0.40) | 0.675 |
|  | >=75 | 0.26 | (-0.34, 0.85) | 0.393 | 0.02 | (-0.18, 0.21) | 0.868 | 0.14 | (-0.17, 0.44) | 0.374 |
| Sex | Female | 0.19 | (-0.05, 0.44) | 0.117 | 0.01 | (-0.06, 0.09) | 0.713 | 0.07 | (-0.05, 0.20) | 0.258 |
| Race | Black | 0.09 | (-0.27, 0.45) | 0.626 | 0.02 | (-0.10, 0.14) | 0.761 | 0.02 | (-0.15, 0.20) | 0.789 |
|  | Other | -0.32 | (-1.24, 0.60) | 0.491 | -0.01 | (-0.30, 0.29) | 0.963 | 0.02 | (-0.38, 0.43) | 0.915 |
| Urbanicity | Urban | -0.25 | (-0.58, 0.08) | 0.143 | -0.06 | (-0.16, 0.04) | 0.261 | -0.13 | (-0.34, 0.08) | 0.209 |

*Effect modifications were incremental effects (i.e. coefficients of the interaction terms) and were evaluated using periods lag0-1 for PM_2.5_, lag0-2 for O_3_, and lag0-2 for NO_2_. Values are percent increase (95% CI) for 10 µg/m^3^ increase in PM_2.5_, 10 ppb in O_3_, and 10 ppb in NO_2_.*

*† The effects of high school and higher-than-high school education were in comparison to lower-than-high school education; the effects of age groups were in comparison to age <45 years; the effects of female gender were in comparison to male; the effects of race of black were in comparison to race of white; the effects of urbanicity (defined as total population density above 25^th^ percentile) were in comparison to rural areas.*
