# Supplementary material for: Short term exposure to air pollution and mortality in the US: a double negative control analysis
Source: Environ Health. 2022 Sep 6;21:81. doi: 10.1186/s12940-022-00886-4 (PMC9446691; doi:10.1186/s12940-022-00886-4)
Supplement: Supplementary file 3 — Additional file 3: Supplementary Table 2. Single lag models for temperature and absolute humidity. [file 12940_2022_886_MOESM3_ESM.docx]

|  | **Temperature** | | | **Humidity** | | |
| --- | --- | --- | --- | --- | --- | --- |
| *Model* | *%* | *95% CI* | *p* | *%* | *95% CI* | *p* |
| *Lag 0* | -0.40 | (-0.54, -0.26) | <0.01 | -472.82 | (-792.80, -152.84) | <0.01 |
| *Lag 1* | -1.08 | (-1.08, -0.79) | <0.01 | -2110.29 | (-2430.53, -1790.06) | <0.01 |
| *Lag 2* | -1.49 | (-1.49, -1.21) | <0.01 | -3226.40 | (-3226.40, -3546.94) | <0.01 |
| *Lag 3* | -1.47 | (-1.47, -1.19) | <0.01 | -3127.71 | (-3127.71, -3448.23) | <0.01 |

*Reported estimates were independent effects without adjustment for the other variable. Values are percent increase (95% CI) for 1 degree Celsius (Kelvin) increase in temperature and 1 g/cm^3^ increase in absolute humidity.*
