# Supplementary material for: Short term exposure to air pollution and mortality in the US: a double negative control analysis
Source: Environ Health. 2022 Sep 6;21:81. doi: 10.1186/s12940-022-00886-4 (PMC9446691; doi:10.1186/s12940-022-00886-4)
Supplement: Supplementary file 4 — Additional file 4: Supplementary Table 3. Effects of Negative Exposure Controls. [file 12940_2022_886_MOESM4_ESM.docx]

|  | **PM2.5 lead (*μ*g /m^3^)** |  |  | **O3 lead (ppb)** |  |  | **NO2 lead (ppb)** |  |  |
| --- | --- | --- | --- | --- | --- | --- | --- | --- | --- |
| *Model* | *%* | *95% CI* | *p* | *%* | *95% CI* | *p* | *%* | *95% CI* | *p* |
| *Three pollutant Model* | -0.36 | (-0.67, -0.11) | <0.01 | 0.19 | (0.01, 0.37) | <0.05 | -0.047 | (-0.23,0.12) | 0.55 |
| *Low Exposure^*^* | -0.32 | (-0.63, 0.003) | 0.05 | 0.27 | (0.07, 0.47) | <0.01 | -0.05 | (-0.23, 0.14) | 0.64 |
| *Cardiovascular^†^* | -0.49 | (-0.97, -0.01) | <0.05 | 0.26 | (-0.05, 0.56) | 0.10 | 0.14 | (-0.16, 0.44) | 0.37 |
| *Respiratory^†^* | -0.38 | (-1.28, 0.52) | 0.41 | -0.57 | (-0.13, -0.003) | <0.05 | -0.42 | (-0.99, 0.16) | 0.16 |
