# Supplementary material for: Short term exposure to air pollution and mortality in the US: a double negative control analysis
Source: Environ Health. 2022 Sep 6;21:81. doi: 10.1186/s12940-022-00886-4 (PMC9446691; doi:10.1186/s12940-022-00886-4)
Supplement: Supplementary file 5 — Additional file 5: Supplementary Table 4. Effects (95% CI) in Single and Double pollutant models. [file 12940_2022_886_MOESM5_ESM.docx]

|  | **PM2.5** | **O3** | **NO2** |
| --- | --- | --- | --- |
| **Single Pollutant Models** | **0.90% (0.58, 1.21)** | **0.33% (0.12, 0.53)** | **0.35% (0.17, 0.53)** |
| **Double Pollutant Models** | **0.85% (0.72, 0.98)** | **0.20% (-0.01, 0.41)** | **NA** |
|  | **NA** | **0.28% (0.07, 0.49)** | **0.34% (0.16, 0.53)** |
|  | **0.78% (0.44, 1.12)** | **NA** | **0.18% (-0.02, 0.37)** |
