# Supplementary material for: Short term exposure to air pollution and mortality in the US: a double negative control analysis
Source: Environ Health. 2022 Sep 6;21:81. doi: 10.1186/s12940-022-00886-4 (PMC9446691; doi:10.1186/s12940-022-00886-4)
Supplement: Supplementary file 6 — Additional file 6. Use of negative outcome and negative exposure controls to obtain estimated effects corrected for bias by omitted confounders. [file 12940_2022_886_MOESM6_ESM.docx]

Appendix 1. Use of negative outcome and negative exposure controls to obtain estimated effects corrected for bias by omitted confounders.

Suppose A is the exposure, Z the negative exposure control, Y the outcome, and W the negative outcome control. Let U be the unmeasured confounder. Assume further that:

$Clogit\left( Y \right)=\beta_{Y0}+\beta_{YA}A+\beta_{YU}U.$ (1)

And

$Clogit\left( W \right)=\beta_{WY0}+\beta_{WU}U$ (2)

since by hypothesis W does not depend on A.

Further assume that the confounder U is related to A and Z by

$$E\left( U \right)=\beta_{U0}+\beta_{UA}A+\beta_{Uz}Z. (3)$$

If we replace U in the first equation by its expectation in the third equation we have

$$Clogit\left( Y \right)=\beta_{Y0}+\beta_{YA}A+\beta_{YU}\left( \beta_{U0}+\beta_{UA}A+\beta_{Uz}Z \right)$$

$$={(\beta}_{Y0}+\beta_{YU}\beta_{U0})+(\beta_{YA}+\beta_{YU}\beta_{UA}) A+\beta_{YU}\beta_{Uz}Z$$

Hence the bias in the estimate of the effect of A is $\beta_{YU}\beta_{UA} .$ If β_UA_=β_UZ_, then that is precisely the coefficient of Z, the negative control in the outcome regression, and subtracting it from the estimate of the coefficient of A in that model $(\beta_{YA}+\beta_{YU}\beta_{UA})$ will recover the true unbiased coefficient of A, β_YA._ Since in our case Z is the identical variable to A, merely a day later, this is not an unreasonable assumption. If the association of the confounder U with A and Z are proportional to each other, but with the same sign, then the magnitude of the bias estimated by the coefficient of the negative control exposure (Z) will be off, but the sign of the bias will be correct.

Next, consider the model for the negative outcome control and again substitute U by its expectation.

$Clogit\left( W \right)=\beta_{WY0}+\beta_{WU}U=(\beta_{WY0}$ +$\beta_{WU}\beta_{U0})+\beta_{WU}\beta_{UA}A+\beta_{WU}\beta_{Uz}Z$

If $\beta_{WU}$, the coefficient of U predicting the negative control outcome is the same as $\beta_{YU}$, the coefficient of U predicting the true outcome, then the coefficient of A in the negative outcome control is the bias in the estimated effect of A on Y due to unmeasured confounding U. And subtracting that coefficient will again correct the estimate. This is a stronger assumption than the previous one since it is less obvious that $\beta_{YU}{=\beta}_{WU}$.

Finally we note that by equation 2, the negative outcome control is a surrogate for U. If we regress W on A and Z, the linear predictor should capture the part of U that is associated with Z and A. Hence, if we add the expected value of the linear predictor as a covariate to the outcome regression for Y, this should adjust for confounding by U. The conditions for this to hold are that the associations are all linear in the exposures, positivity, that the negative outcome control is independent of exposure conditional on the measured and unmeasured confounders, and that the negative exposure control is independent of the outcome and negative outcome control. We have added this analysis to the manuscript.
